# Effectiveness and usage of a decision support system to improve stroke prevention in general practice: A cluster randomized controlled trial

**DOI:** 10.1371/journal.pone.0170974

**Published:** 2017-02-28

**Authors:** Derk L. Arts, Ameen Abu-Hanna, Stephanie K. Medlock, Henk C. P. M. van Weert

**Affiliations:** 1 Academic Medical Centre, Department of General Practice Amsterdam, The Netherlands; 2 Academic Medical Centre, Department of Medical Informatics, Amsterdam, The Netherlands; University of Glasgow, UNITED KINGDOM

## Abstract

**Background:**

Adherence to guidelines pertaining to stroke prevention in patients with atrial fibrillation is poor. Decision support systems have shown promise in increasing guideline adherence.

**Aims:**

To improve guideline adherence with a non-obtrusive clinical decision support system integrated in the workflow. Secondly, we seek to capture reasons for guideline non-adherence.

**Design and setting:**

A cluster randomized controlled trial in Dutch general practices.

**Method:**

A decision support system was developed that implemented properties positively associated with effectiveness: real-time, non-interruptive and based on data from electronic health records. Recommendations were based on the Dutch general practitioners guideline for atrial fibrillation that uses the CHA_2_DS_2_-VAsc for stroke risk stratification. Usage data and responses to the recommendations were logged. Effectiveness was measured as adherence to the guideline. We used a chi square to test for group differences and a mixed effects model to correct for clustering and baseline adherence.

**Results:**

Our analyses included 781 patients. Usage of the system was low (5%) and declined over time. In total, 76 notifications received a response: 58% dismissal and 42% acceptance. At the end of the study, both groups had improved, by 8% and 5% respectively. There was no statistically significant difference between groups (Control: 50%, Intervention: 55% P = 0.23). Clustered analysis revealed similar results. Only one usable reasons for non-adherence was captured.

**Conclusion:**

Our study could not demonstrate the effectiveness of a decision support system in general practice, which was likely due to lack of use. Our findings should be used to develop next generation decision support systems that are effective in the challenging setting of general practice.

## Introduction

The burden of atrial fibrillation (AF) increases year by year in societies with aging populations, such as Western Europe and the United States [1–3]. This burden is largely due to the five-fold increased risk for stroke that is associated with AF [4]. There is ample evidence that the efficacy of oral anticoagulation (OAC) in stroke prevention is excellent [5–7]. Nonetheless overtreatment should be avoided, as this can result in hemorrhaging, mainly intracerebral and gastrointestinal. Therefore, treatment strategies for stroke prevention have been optimized over recent years, and clinical practice guidelines describing these treatment strategies are publicly available [8]. However, adherence to these guidelines remains poor [9–11]. This non-adherence is mainly related to underuse of OAC in patients with medium to high stroke risk. Two important reasons for OAC underuse are 1) the complexity of the decision rules used and 2) physicians’ concerns with the bleeding risk associated with OAC. However, the benefit of stroke prevention greatly outweighs the risk of bleeding due to OAC, and OAC should therefore not be withheld when indicated [6, 12].

Studies have shown that clinical decision support systems (CDSSs) have the potential to improve guideline adherence [13–15], as these systems can make physicians aware of the guidelines when they lack the appropriate knowledge and eliminate inappropriate use of clinical decision rules. The overall effectiveness of CDSS on mortality has not been established, but a recent review did find a moderate improvement (RR = 0.82; 95% CI = [0.68, 0.99]) in morbidity outcomes [16]. CDSSs therefore present a possible solution for the issue of guideline non-adherence in stroke prevention among AF patients.

CDSS effectiveness varies by study and domain, and several studies have tried to determine what factors predict success or failure,[17, 18] [19] although it is hard to pinpoint these [20].We have developed a CDSS that implements properties that appeared to have a positive effect on CDSS effectiveness, most notably: “implementing decision support as part of the workflow”, “during the time of decision making” and “optimized human-computer interface”[18, 21]. Too many alerts will tend to result in all alerts being ignored, a phenomenon known as “alert fatigue” [22]. Given the possible adverse effects of “alert fatigue” and interruption [23], we considered the optimal interface to be one which minimized these effects.

In this study we investigate the effectiveness of our CDSS as measured by general practitioners’ adherence to the Dutch GP guideline for patients with atrial fibrillation. A secondary objective of this study is to gain insight into reasons for deviations from the guideline and to adjudicate these reasons with peers.

We hypothesized that the implementation of a non-obtrusive CDSS, integrated in the GP’s workflow, will increase guideline adherence. Secondly, we expect GPs will often have valid reasons for guideline non-adherence.

This trial is registered with the Dutch Trial Register under registration number 3570. http://www.trialregister.nl/trialreg/admin/rctview.asp?TC=3570

## Materials and methods

The trial protocol for this study, which was dubbed the “Expert-AF” trial, has been published elsewhere [24]. Below, we will provide a short overview of the most important aspects of the methodology used.

### Design

A cluster-randomized controlled design was used. Randomization was done at the GP practice level to reduce contamination bias. GPs were allocated into one of the following three groups:

1. Received no messages (control group)
2. Received messages that could be declined without documenting justification (intervention 1)
3. Received messages that could only be declined if justification was documented (intervention 2)

**Fig 1:**
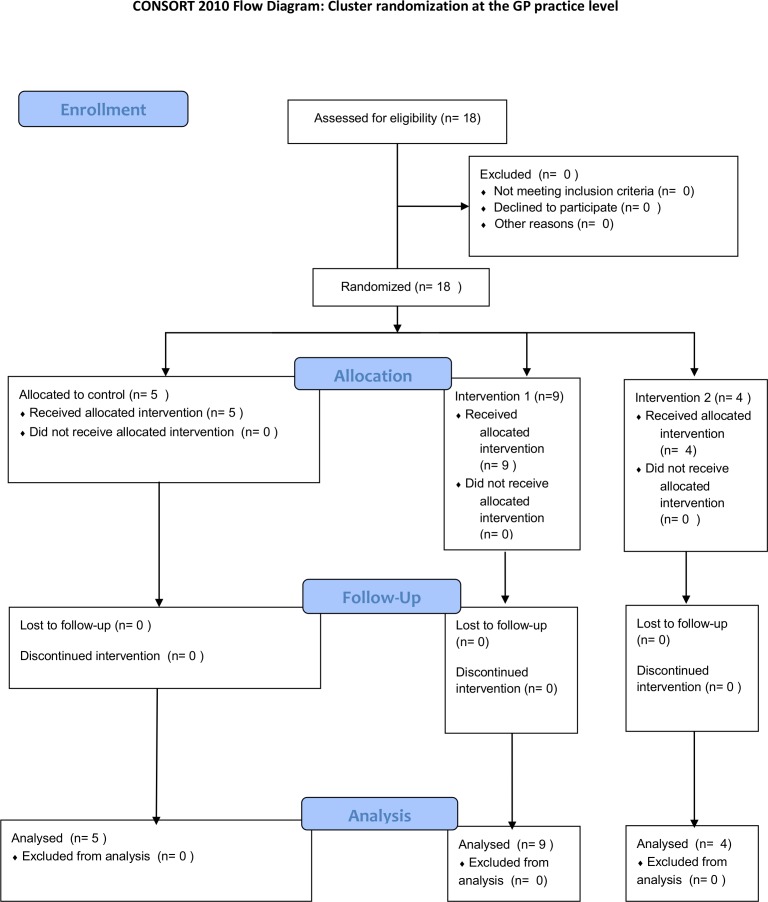
The CONSORT flow diagram.

The planned allocation ratio was 2:1:1 respectively (Fig 1). Randomization was performed in the statistical environment R [25] using the ‘sample’ function to create a random sequence from the list of GP practices by DA. GPs were aware that they were allocated to a variant of the system, but were blinded as to their allocation and how the variants differed.

### Regulatory aspects

The medical ethics committee of the AMC deemed this study exempt from ethical approval because the intervention is a way of providing valid medical information to GPs and does not change medical treatment or use invasive procedures. Furthermore, only anonymized patient data was used for analysis. All GPs consented to participation in the trial.

### Population

We planned to include GP practices in the Netherlands that used the electronic health record (EHR) for which we developed a decision support plugin (an extra piece of software providing feedback that is not an integral part of the main system). All patients with AF (both incident and pre-existing) who had been in contact with their GPs during the length of the trial were evaluated. The GPs use ICPC-coded diagnoses for both care and reimbursement, thus we considered a patient to have AF if a coded diagnosis (‘K78’) for AF was documented [26]. This method was chosen to exclude care avoiders (patients who actively avoid contact with their GP despite their condition) that could bias results.

### Intervention

**Fig 2:**
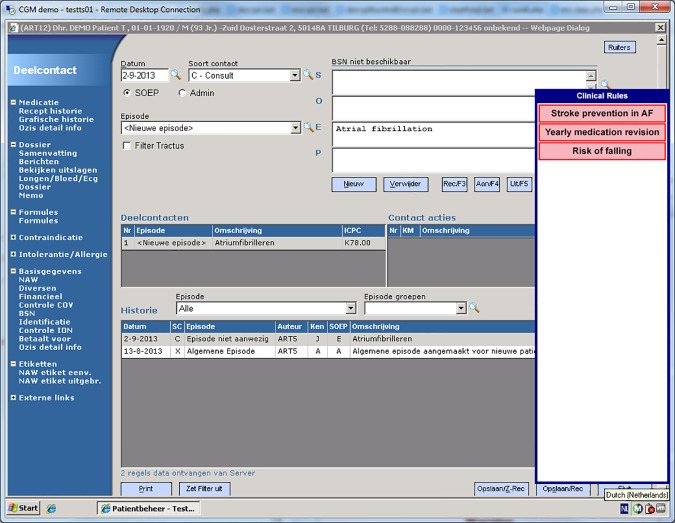
The notification window in its expanded state, showing three notifications, with the AF notification at the top of the list that includes other notifications originating from other clinical rules.

**Fig 3:**
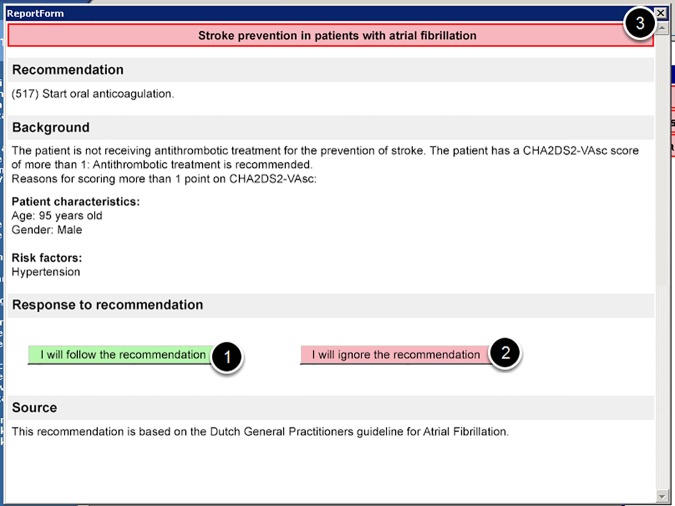
The popup after clicking on a notification, containing background information, an actionable recommendation and response buttons that allowed the GP to either accept (1) or decline (2) the advice.

We developed a CDSS for a single EHR system. This CDSS was automatically activated using event-based triggers, and responded to actions of the GP in real time [24]. Our study ran concurrently with the *ICOVE trial* that contained 15 decision rules per randomization group [27]. For each decision rule that was not satisfied, notifications were shown in a floating notification window. The window could contain multiple notifications if multiple rules applied to the patient. Notifications contained a short (1- to 3-word) title (Fig 2). By clicking on a notification, a popup appeared that contained: background information, an actionable recommendation and two response buttons that allowed the GP to either accept or decline the advice (Fig 3). Accepting or declining was logged by the plugin but had no effect on the EHR. Due to the fact that there were other notifications present in the system, GPs were likely unaware of their allocation. The Expert-AF notification was positioned at the top of the notification window. For intervention group 2 there was a small free text box that required the GP to enter a reason for deviating from the recommendation. The plugin was only active for GPs.

### Training and support

GPs were trained using three methods: a live demonstration, a PDF manual and a video demonstration. One live demonstration per GP practice was provided. Additionally, a detailed manual of the system was distributed in PDF format among all participants. Lastly, GPs were made aware per email of a 10-minute video demonstration of the system available online. Support was available during office hours via email and phone.

### Decision rule

The computerized decision rule used in the Expert-AF system was based on the Revised Dutch GP guideline for atrial fibrillation, authored by the Dutch College of General Practitioners [28]. This guideline uses the CHA_2_DS_2_-VASc score, which consists of 7 variables to predict risk of stroke [29]. Patients can score from 0 to 9 points. All patients with a score >1 have an indication for OAC according to the current GP guideline. These same recommendations are made in the 2012 European Society of Cardiology guideline on atrial fibrillation [8]. The HAS-BLED bleeding risk score [30] was not yet formally introduced in the Dutch GP guideline for AF, and thus not included in the decision rule. However previous large bleeds, untreated hypertension, kidney failure and thrombotic disorders were included as contra-indications for VKA.

### Baseline data collection

Baseline measurements were performed to determine the proportion of patients with AF who were treated in accordance with the guideline. Guideline adherence was determined by automatically evaluating the computerized decision rule on all patients in an anonymized copy of the GP’s EHR databases.

### Data collection during and after the study

Every action that related to the plugin was stored. Anonymized patient files were saved to confirm the system functioned properly and to determine what triggered the notifications. Guideline adherence by the participating GPs was evaluated using an anonymized copy of the GP EHR databases, which was made after the trial was completed. The trial was conducted from October 1st, 2013 to September 1st, 2014, resulting in 240 active workdays.

### Outcomes

We defined the primary outcome as the difference in the proportion of patients with AF treated in accordance with the guideline between the intervention and control groups.

A secondary outcome of the study was the reasons GPs provided for deviating from the guideline and the manner in which they responded to required justification (intervention 2).

### Analysis

A power analysis accounting for clustering resulted in a required sample size of 500 patients for an effective sample size of 300 [24].

A chi-square test was used to compare between-group differences at baseline and differences in adherence rates post-study. Additionally, a mixed effects logistic regression model was used with the GP practice as random effect to correct for clustering at the practice level and baseline adherence.

Intervention 2 was an extension of intervention 1; the only difference between the two was the addition of a free text box that required a reason for ignoring recommendation. For the all quantitative analyses and plots, these groups were merged to increase statistical power. Analyses were performed using R [25].

## Results

For the baseline analyses 731 patients with AF were included (Table 1). In total, 781 patients with AF were in contact with the GP practice during the trial and included in the analysis. These patients were seen by 39 GPs in 19 GP clusters.

**Table 1:** Population characteristics and guideline adherence before and after the study.

	Baseline control	Baseline intervention		Post-study control	Post-study intervention	
**N**	235	496		259	522	
**Mean age (SD)**	74.61 (13.63)	72.13 (12.46)	P = 0.08	73.73 (14.7)	72.79 (12.61)	P = 0.52
**Mean CHA**_**2**_**DS**_**2**_**-VAsc (SD)**	3.06 (1.8)	3.00 (1.72)	P = 0.52	2.27 (0.82)	2.25 (0.86)	P = 0.13
**% CHA**_**2**_**DS**_**2**_**-VAsc > 1**	80	78		76	76	
**% on OAC**	40	48	P = 0.05	51	60	P = 0.02
**% adherence**	42	50	P = 0.04	50	55	P = 0.23

### Usage

**Fig 4:**
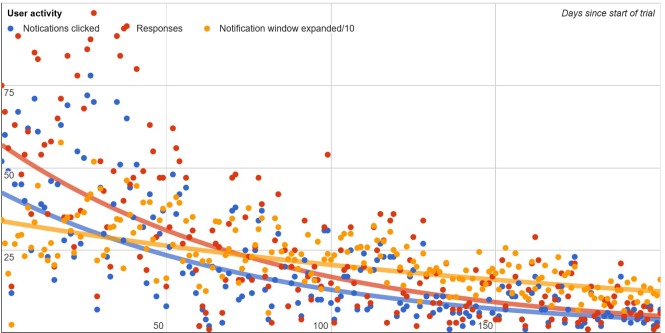
Usage of the CDDS plugin over time. Blue dots indicate clicked notifications, red dots indicate responses to recommendations (clicking accept or decline), orange dots indicate the number of times the GP hovered over the notification window with their mouse regardless of whether the notification was opened.

**Fig 5:**
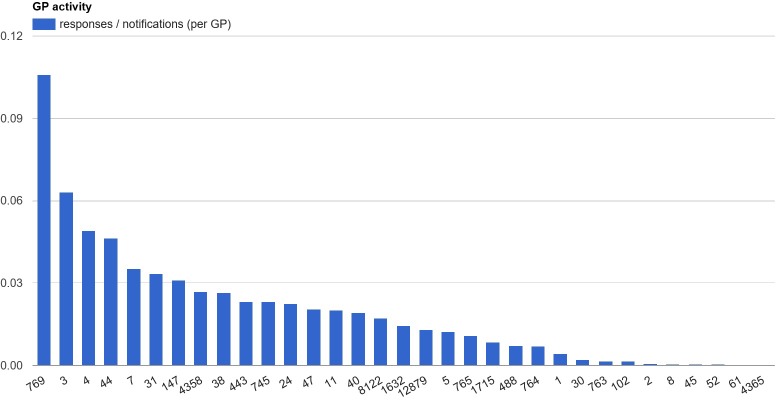
User activity Average usage (responses / notification) per general practitioner.

Table 2 contains usage statistics. The notification was shown 3848 times, and clicked 188 times to open the pop-up with the information and advice (5%). Usage over time declined, with the most activity in the first month, and a steady decline of usage over the following 8 months (Fig 4). In total, GPs actively responded to 76 notifications by either dismissing them (N = 44, 58%) or indicating they would follow the advice (N = 32, 42%). Some GPs did not click on the notification at all, while others clicked nearly all of them (Fig 5).

**Table 2:** Usage of the system during the 9 months.

**Notifications shown**	3848
**Notifications clicked**	188
**Responses to recommendations**	76
**Clicks per notification**	0.05
**Clicks per consultation**	0.07
**Days usage**	240
**Avg. # notifications per workday**	16
**Avg. # notifications per workday per GP**	0.45

In the second intervention group, GPs clicked on twenty-two notifications and accepted two. Five notifications were declined. Four reasons for declining were related to the plugin not being able to detect Warfarin as active medication or not related to the guideline, thus only one reason for guideline non-adherence was presented, which was insufficient for further analysis.

### Effectiveness

Guideline adherence, defined as the percentage of patients treated in accordance with the guideline, differed between the control and intervention groups at baseline (Control: 42%, Intervention: 50% chisq P = 0.04). At the end of the study, both groups had improved, by 8% and 5% respectively. There was no statistically significant difference between groups (Control: 50%, Intervention: 55% chisq P = 0.23) (Table 1). Clustered analysis revealed similar results (P = 0.21); correcting for baseline adherence did not alter these results (P = 0.25).

## Discussion

### Summary

We evaluated the effectiveness of a real-time CDSS in general practice, specifically for increasing adherence to antithrombotic guidelines for atrial fibrillation. We did not find a significant difference in guideline adherence between the intervention and control groups.

### Strengths and limitations

Strengths of this study included the decision support system feedback characteristics that built on evidence pertaining to effectiveness of decision support systems [17, 18]. We implemented a real-time CDSS that supported the GP during their contacts with patients by showing alerts in a non-obtrusive way. Our trial implemented many of the features that we should expect from decision support in the future. We reduced contamination by using clustered randomization. Lastly we studied a topic (prevention of stroke), that is considered highly relevant by the participating GPs.

However, this study was hampered in several ways. First, the vendor of the GP system that implemented the CDSS plugin stopped supporting it, thereby preventing us from enrolling more GPs in our trial. As initially, more GPs were included in the intervention arm, this lead to asymmetric randomization groups, reducing potential power of the study. Nonetheless the calculated sample size (n = 500) was reached, with more than 250 inclusions in each group post study. We would have preferred to develop a generic system that worked on any platform but were due to funding and organizational restrictions we were limited to working with a single vendor.

Lastly, the new guideline for the management of AF by the Dutch College of General Practitioners was introduced during the start of the trial. Combined with the fact that all GPs were trained in use of the system, it is likely there was a teaching effect, or at least a substantial increase in awareness of OAC under prescribing in patients with AF. This might have resulted in increased adherence in both groups. We selected guideline adherence as primary outcome, but perfect guideline adherence is undesirable as most guidelines do not account for complex patients with multi-morbidity and physicians often have good reasons for deviating from a guideline [31]. Thus, using guideline adherence as outcome measure might have decreased the potential room for improvement of our intervention.

### Comparison with existing literature

#### Guideline adherence

Guideline adherence was poor both before and after the study, which is in line with other studies that specifically investigated this topic [9, 11, 32]. Guideline non-adherence consisted of both under- and overtreatment, which was also present in other studies investigating OAC use [9, 11, 33]. Nonetheless, the increased OAC use in both groups could indicate awareness of OAC underuse is increasing.

#### Effectiveness

We attribute the lack of effectiveness mainly to low usage (measured by interaction with the notifications), which had many reasons. These will be discussed in detail in a separate qualitative system evaluation. Briefly, barriers were mainly related to lack of time, too many alert notifications and limitations of the system’s functionality. Participants in acknowledged the potential of CDSSs for the future of healthcare, but implementing these systems in daily practice for multiple domains remains challenging. Alert prioritization, user customization, tight EHR integration and strict selection of alerts might improve CDSS effectiveness. Eccles et. al. found similar low usage rates and attributed the limited effectiveness of their system to this lack of use [34]. Lugtenberg et. al. recently published results on a process evaluation of ‘NHGDoc’, an EHR-integrated decision support system that was implemented in 65% of GP practices in the Netherlands. Usage of the system was as low as 0.24%, and it also did not lead to changes in outcomes [35]. A recent trial by Cook et. al. that attempted to improve prescription in AF by way of a CDSS also failed to show effectiveness of their intervention [36]. While Cook et al did not report on usage, low usage may have also hampered their study. All four studies (including ours) attempted to provide the user with a non-obtrusive system, integrated in daily workflow. To provide more insight on when CDSS might be more effective, we performed a sub-group analysis on patients with incident AF. Although this sample was too small to be adequately powered, there was a trend towards effectiveness in this sub-group (P = 0.09 vs P = 0.23), which could indicate that GPs might be more inclined to follow CDSS based recommendations when first treating a patient. However, this trend could also be due to the difference between the quality of old and new data or other biases, and should be investigated in detail in future studies.

It is still unclear what can be considered ‘optimal timing’ in the busy daily practice. Its non-obtrusive nature may have been disadvantageous in a setting where patients, staff, e-mails and other matters compete for the GP’s attention. Recent studies have indicated that systems which force the user to respond to the alert are more effective [19], although clearly only a limited number of alerts can be presented in this way. Furthermore, physicians do not always agree with the guidelines these systems are based on,3 or have (valid) reasons for non-adherence [31, 37, 38]. The number of declined recommendations (N = 44, 54%) we found in our study suggests that this is at least part of the problem. ‘In terms of the recently-described “Two Stream Model”[20], the lack of use can be attributed mainly to data quality problems and sub-optimal presentation.

### Implications for research

Guideline adherence in both the intervention and control groups increased during this trial investigating the effectiveness of a CDSS for stroke prevention. Our study could not demonstrate the effectiveness of our intervention, which was likely due to lack of use. The implementation of multi-domain CDSSs in clinical practice is challenging and future studies should investigate further improvements to facilitate effectiveness in a real-world setting. Stroke prevention in patients with AF is a field where much can be gained by following guidelines, and we therefore urge other researchers to dedicate their efforts to investigate how to effectively increase system use and guideline adherence.

## Supporting information

S1 Checklist The Consort checklist for this study. (DOC)

S1 Dataset The cleaned dataset used for this paper. (CSV)

S1 Protocol The study protocol for the ExpertAF study. (DOCX)
